# Soybean-Derived Tripeptide Leu–Ser–Trp (LSW) Protects Human Vascular Endothelial Cells from TNFα-Induced Oxidative Stress and Inflammation via Modulating TNFα Receptors and SIRT1

**DOI:** 10.3390/foods11213372

**Published:** 2022-10-26

**Authors:** Hongbing Fan, Khushwant S. Bhullar, Zihan Wang, Jianping Wu

**Affiliations:** Department of Agricultural, Food and Nutritional Science, 4-10 Ag/For Building, University of Alberta, Edmonton, AB T6G 2P5, Canada

**Keywords:** soybean, bioactive peptides, endothelial cells, inflammation, superoxide, malondialdehyde, NF-κB signaling, MAPK signaling, sirtuin-1

## Abstract

Soybean is a rich source of high-quality proteins and an excellent food source of bioactive peptides. A tripeptide, Leu–Ser–Trp (LSW), was previously identified from soybean as an angiotensin-converting enzyme inhibitory peptide. In the present work, we further studied its antioxidant and anti-inflammatory activities in human vascular endothelial cells (EA.hy926) and elucidated the mechanisms underlying these biological activities. In tumor necrosis factor alpha (TNFα)-stimulated EA.hy926 cells, LSW significantly inhibited oxidative stress, both reduced superoxide and malondialdehyde levels (*p* < 0.001), owing to its free-radical-scavenging ability. LSW treatment also mitigated the elevated protein expression of vascular adhesion molecule-1 (*p* < 0.001) and cyclooxygenase 2 (*p* < 0.01) via inhibition of NF-κB and p38/JNK signaling, respectively. Additionally, LSW also inhibited the endogenous formation of TNFα and attenuated the expression of its two receptors in EA.hy926 cells. Furthermore, LSW upregulated sirtuin-1 level, which partially contributed to its anti-inflammatory activity. These results demonstrate the multiple roles of LSW in ameliorating vascular endothelial oxidative stress and inflammatory responses, which support its uses as a nutraceutical or functional food ingredient for combating endothelial dysfunction and cardiovascular diseases.

## 1. Introduction

The vascular endothelium forms the inner monolayer of the blood vascular wall and plays critical roles in the regulation of vascular homeostasis [1,2,3]. Excessive and sustained oxidative stress and inflammatory responses in the vascular endothelium lead to endothelial dysfunction, thus contributing to the onset and augmentation of cardiovascular diseases [4,5,6]. However, increasing evidence shows that oxidative stress and inflammation can be mitigated and delayed by dietary intervention; moreover, dietary intake of natural bioactives can prevent possible side effects due to long-term pharmacological treatment [7].

Food-derived bioactive peptides have attracted substantial attention due to the application and efficacy in preventing and treating various chronic diseases [8,9]. These peptides are primarily derived from various animal (e.g., milk, meat, fish, and egg) and plant (e.g., cereal, legume, and nut) protein sources [10]. Among these natural sources, soybean is a source of numerous bioactive peptides with a wide range of health benefits. Soybean is the most widely planted legume in the world and a key cash crop in many countries across the globe. Soybean has been considered a healthy food, since it is a rich source of dietary proteins that contains amino acid composition akin to that of animal proteins. Indeed, soybean proteins have been used to produce bioactive peptides with numerous beneficial health properties [11,12,13]. In our previous study, a tripeptide, LSW, was purified from soybean glycinin via enzymatic hydrolysis using thermolysin, and it exhibited strong potency in inhibiting angiotensin-converting enzyme activity [14]. Later, peptide LSW was found to be transported across the Caco-2 cell monolayer intact in vitro, indicating its capacity to pass through small intestinal epithelium and exerting biological effects in its innate form [15]. Recently, LSW was also reported to reduce angiotensin-II-induced secretion of extracellular vesicle from vascular smooth muscle cells, which could indirectly prevent vascular endothelial damage [16]. However, whether LSW itself could protect vascular endothelial cells against cellular stress remains elusive. Thus, the overall objectives of the current study were to explore the anti-inflammatory and antioxidant activities of LSW and elucidate the cellular mechanisms underlying these two biological activities in vascular endothelial cells.

## 2. Materials and Methods

### 2.1. Materials

Dulbecco’s modified Eagle’s medium (DMEM), 4-(2-68 hydroxyethyl)-1-piperazineethanesulfonic acid (HEPES), 0.25% (*w*/*v*) trypsin-0.53 mM EDTA, fetal bovine serum (FBS), penicillin-streptomycin (pen-strep), and nonessential amino acids (NEAA) were purchased from Gibco Invitrogen (Burlington, ON, Canada). Triton-X-100 and Dithiothreitol (DTT) were obtained from Sigma Aldrich (St. Louis, MO, USA). Dihydroethidium (DHE) was purchased from Biotium (Fremont, CA, USA). TNFα was obtained from R&D Systems (Minneapolis, MN, USA). Human vascular endothelial EA.hy926 cells were obtained from American Type Culture Collection (CRL-2922; Manassas, VA, USA). Tripeptide LSW (>98% purity) was synthesized by GenScript Biotech Corp. (Piscataway, NJ, USA).

### 2.2. Culturing Protocol of Human Vascular Endothelial EA.hy926 Cells

EA.hy926 cells (at passages 5–8) were cultured by referring to the procedures described in Fan et al. [17]. Cells were grown in complete media in 75 cm^2^ flasks (DMEM containing 10% FBS, 1% NEAA, 25 mM HEPES, and 1% pen-strep) in an incubator (37 °C, 100% humidity, 5% CO_2_); the media was changed every 3 days. For all experiments conducted, cells were first seeded onto culture plates and grown in the complete media, which were then replaced with quiescing media (same as the recipe of the complete media but contained 1% FBS) for various treatments, except for the cytotoxicity test.

### 2.3. Cytotoxicity Assay

Cell viability was measured using alamarBlue assay (Thermo Fisher Scientific, Burlington, ON, Canada), as previously described in Fan et al. [17]. The cells were grown on a 96-well plate, treated with peptide LSW at various concentrations over 24 h in the complete media, which were then replaced with alamarBlue solution for another 4 h (protected from direct light). The resultant solution was transferred to an opaque 96-well plate for the detection of fluorescence signal on a SpectraMax M3 plate reader (emission wavelength: 590 nm; excitation wavelengths: 560 nm) (Molecular Devices, CA, USA). A group (without any treatment) was used as the control.

### 2.4. Western Blotting

After treatment, cells were first washed with cold PBS and were then lysed using boiling Laemmli’s buffer (containing 50 mM DTT and 0.2% Triton-X-100). Lysates were loaded and run in a 9% separating gel, followed by being transferred onto a nitrocellulose membrane, blocked with bovine serum albumin, and incubated with primary and secondary antibodies [18]. The effect of LSW treatment on inflammatory proteins and TNFα receptors was studied after 6 h of TNFα stimulation. Protein bands of COX2 (ab15191, Abcam, ON, Canada), VCAM-1 (sc-8304, Santa Cruz Technology, Dallas, TX, USA), TNFα receptor 1 (TNFR1; sc-8436, Santa Cruz Technology), TNFα receptor 2 (TNFR2; sc-8041, Santa Cruz Technology), and sirtuin-1 (SIRT1; 9475, Cell signaling Technology, Danvers, MA, USA) were normalized to GAPDH (ab8245, Abcam). The effect of LSW treatment on intracellular signaling was studied after 15 min of TNFα stimulation, including nuclear factor erythroid-2-related factor 2 (Nrf2), nuclear factor kappa light polypeptide enhancer in B-cells (NF-κB), and mitogen-activated protein kinases (MAPK) signaling. Protein bands of phospho-Nrf2 (Ser40) (PA5-67520, Invitrogen), phospho-c-Jun N-terminal kinase (JNK) (p-JNK, Thr183/Tyr185) (MAB1205; R&D Systems), phospo-p38 (p-p38, Thr180/Tyr182) (9216; Cell signaling Technology), and phospo-p65 (p-p65, Ser536) (3033; Cell signaling Technology) were normalized to their respective total forms t-Nrf2 (PA5-27882, Invitrogen), t-JNK (MAB1387; R&D Systems), t-p38 (9212; Cell signaling Technology), and t-p65 (sc-8008; Santa Cruz Technology); that of nuclear factor kappa light polypeptide enhancer in B-cells inhibitor alpha (IκBα; 9242, Cell signaling Technology) was normalized to GAPDH. The fluorescence secondary antibodies were obtained from Licor Biosciences (Lincoln, NE, USA); bands were visualized using Licor BioImager, and the intensity was quantified using Image Studio Lite 5.2 (Licor Biosciences).

### 2.5. Measurement of TNFα in EA.hy926 Cells

The cells were grown on a 6-well plate until reaching ~80% confluence. After treatment, cells were trypsinized and centrifuged at 1000× *g* for 5 min. The pellets were washed using cold PBS, centrifuged again, and then lysed using radioimmunoprecipitation assay (RIPA) buffer (Abcam) in ice for 30 min. Cell lysates’ TNFα levels were analyzed using the Elisa kit (ab181421, Abcam) according to the manufacturer’s manual.

### 2.6. Quantitative Reverse Transcription Polymerase Chain Reaction (qRT-PCR)

qRT-PCR was performed to quantify the messenger RNA expression [19]. Briefly, RNA was extracted using TRIzol^TM^ reagent (Thermo Fisher Scientific) and quantified using Nanodrop (Thermo Fisher Scientific); only high-quality RNA samples were selected to synthesize complementary DNA (cDNA) through reverse transcription. qRT-PCR was performed using Fast SYBR green Master Mix on a StepOnePlus^TM^ Real-Time PCR System (Applied Biosystems, Burlington, ON, Canada). Primers of SIRT1 and GAPDH were TAGACACGCTGGAACAGGTTGC (F)/CTCCTCGTACAGCTTCACAGTC (R) and GTCTCCTCTGACTTCAACAGCG (F)/ACCACCCTGTTGCTGTAGCCAA (R), respectively (Thermo Fisher Scientific). Fold change of the target gene was calculated using GAPDH (housekeeping gene) and normalized to the control group (without treatment), and expressed as cycle of threshold as described in Fan et al. [19].

### 2.7. Superoxide Detection and Lipid Peroxidation Assay

Detection of superoxide was performed by DHE staining assay, as previously depicted in Fan et al. [17]. Malondialdehyde (MDA) level in EA.hy926 cells was measured using lipid peroxidation assay kit (Abcam) [17].

### 2.8. Statistical Analysis

All analyses were performed with at least 3 independent experiments. Data were expressed as means ± standard errors of means (SEMs) and were subjected to one-way ANOVA followed by Tukey’s post hoc test using GraphPad Prism 9 (San Diego, CA, USA). The *p* values of <0.05 and <0.01 were considered statistically significant and highly significant, respectively.

## 3. Results

### 3.1. LSW Does Not Cause Cytotoxicity in TNFα-Stimulated EA.hy926 Cells

**Figure 1:**
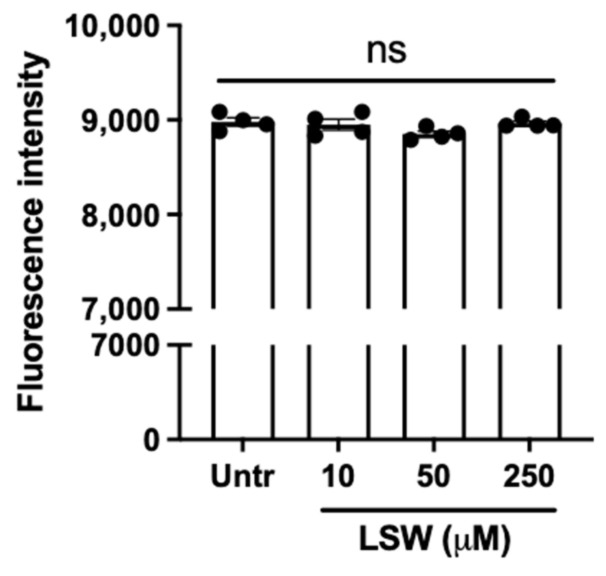
Cell viability upon LSW treatment. EA.hy926 cells were treated with different concentrations of LSW over 24 h before being analyzed by the alamarBlue cell viability assay. Untr., untreated; ns, not significant (*p* > 0.05).

Effect of peptide LSW on the viability of EA.hy926 cells was assessed prior to studying its antioxidant and anti-inflammatory activities. Figure 1 illustrates that incubation of LSW at the concentration of 10–250 μM over 24 h had no adverse impact on the cell viability (*p* > 0.05). Since a lower concentration (50 μM) was used for all the experiments, peptide LSW was considered not toxic against EA.hy926 cells in the present work.

### 3.2. LSW Reduces Oxidative Stress in TNFα-Stimulated EA.hy926 Cells

**Figure 2:**
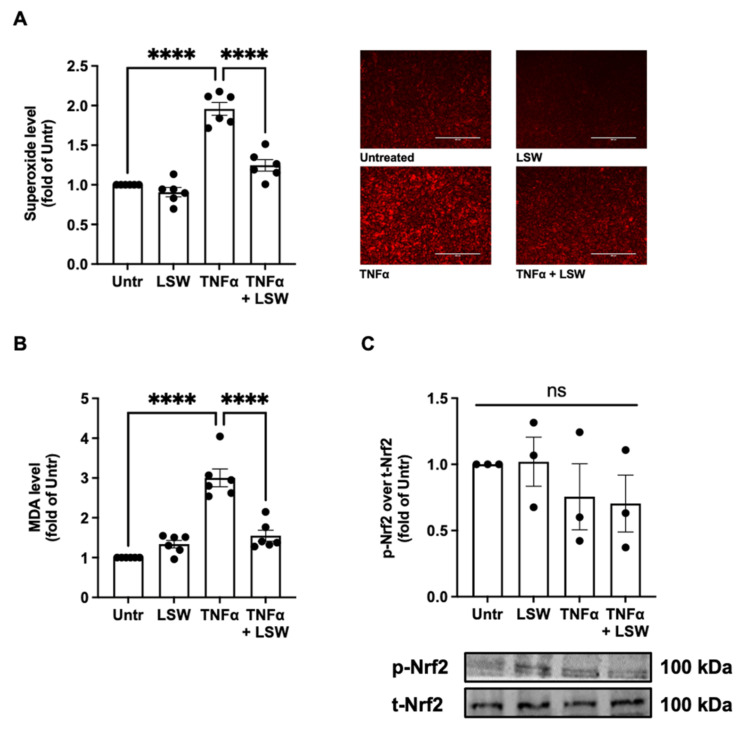
Effect of LSW treatment on TNFα-induced oxidative stress in EA.hy926 cells. The cells were treated with LSW (50 μM) for 18 h before TNFα (10 ng/mL) stimulation for another 30 min followed by superoxide detection (**A**), or for another 6 h followed by malondialdehyde (MDA) measurement (**B**) and protein expression of Nrf2 (**C**). Protein bands of phosphorylated Nrf2 (p-Nrf2) were normalized to their total form (t-Nrf2). Data were normalized to the untreated group (Untr). ****, *p* < 0.0001; ns, not significant.

Figure 2A illustrates the effect of LSW treatment on TNFα-induced superoxide generation in EA.hy926 cells. TNFα induced superoxide generation (*p* < 0.0001) in EA.hy926 cells, while LSW treatment significantly inhibited the surged superoxide level (*p* < 0.0001). Additionally, LSW treatment inhibited MDA formation in TNFα-induced EA.hy926 cells (*p* < 0.0001), indicating a lowered lipid oxidation (Figure 2B). LSW almost abolished TNFα-induced superoxide formation and lipid oxidation, indicating its strong antioxidant effect in EA.hy926 cells. However, LSW treatment did not enhance the phosphorylation of Nrf2 (Figure 2C), suggesting that the antioxidant activity of LSW was independent of the Nrf2 pathway.

### 3.3. LSW Ameliorates Inflammation in TNFα-Stimulated EA.hy926 Cells

**Figure 3:**
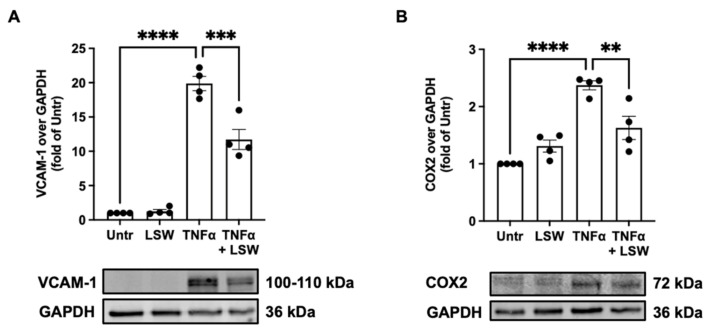
Effect of LSW treatment on TNFα-induced inflammation in EA.hy926 cells. The cells were treated with LSW (50 μM) for 18 h before TNFα (10 ng/mL) stimulation for 6 h, followed by detecting the protein expression of VCAM-1 (**A**) and COX2 (**B**) expression by Western blotting. Protein bands were normalized to GAPDH. Data were normalized to the untreated group (Untr). **, *p* < 0.01; ***, *p* < 0.001; ****, *p* < 0.0001.

Effect of LSW treatment on TNFα-induced inflammation in EA.hy926 cells was studied. Upon stimulation with TNFα, both VCAM-1 and COX2 expressions were increased (*p* < 0.0001), whereas LSW treatment partially inhibited TNFα-stimulated VCAM-1 (*p* < 0.001) and COX2 (*p* < 0.01) expressions in EA.hy926 cells (Figure 3).

### 3.4. LSW Inhibits NF-κB and p38/JNK MAPK Signaling in TNFα-Stimulated EA.hy926 Cells

**Figure 4:**
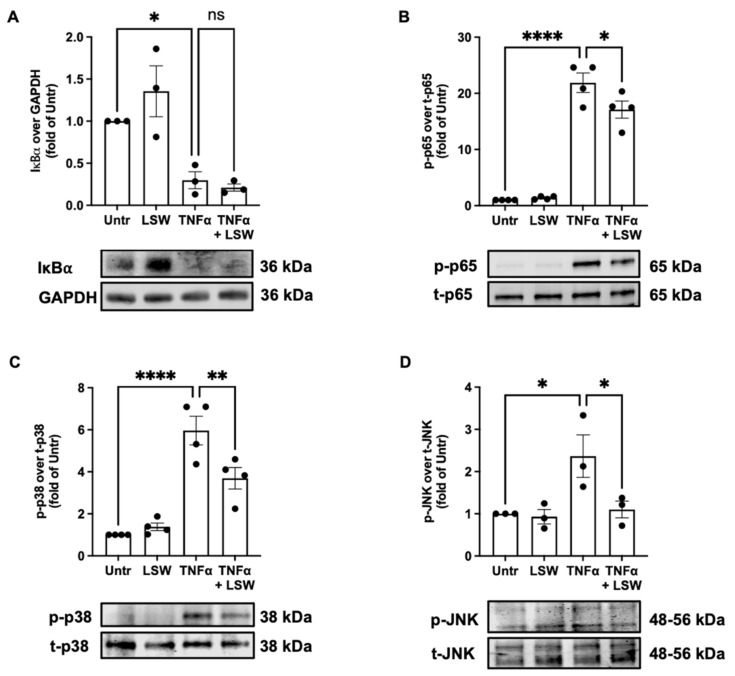
Effect of LSW treatment on TNFα-mediated NF-κB and p38/JNK signaling in EA.hy926 cells. The cells were treated with LSW (50 μM) for 18 h before TNFα (10 ng/mL) stimulation for 15 min, followed by the detecting the protein expression of IκBα (**A**), p65 (**B**), p38 (**C**), and JNK (**D**) by Western blotting. Protein bands of IκBα were normalized to GAPDH; bands of the phosphorylated p65, p38, and JNK were normalized to their total forms. Data were normalized to the untreated group (Untr). *, *p* < 0.05; **, *p* < 0.01; ****, *p* < 0.0001, ns, not significant.

Effect of LSW treatment on TNFα-mediated intracellular signaling, including NF-κB (p65) and MAPK (p38 and JNK) signaling, was analyzed. TNFα triggered transient degradation of IκBα and phosphorylation of p65, whereas LSW treatment partially inhibited the phosphorylation of p65 (*p* < 0.05), without affecting the degradation of IκBα (*p* > 0.05) (Figure 4). Additionally, LSW treatment partially inhibited TNFα-induced phosphorylation of p38 and JNK. These results demonstrate that NF-κB and p38/JNK MAPK signaling underlie LSW’s beneficial role in attenuating endothelial inflammation.

### 3.5. LSW Reduces TNFα/TNFR1/TNFR2 Expressions in TNFα-Stimulated EA.hy926 Cells

**Figure 5:**
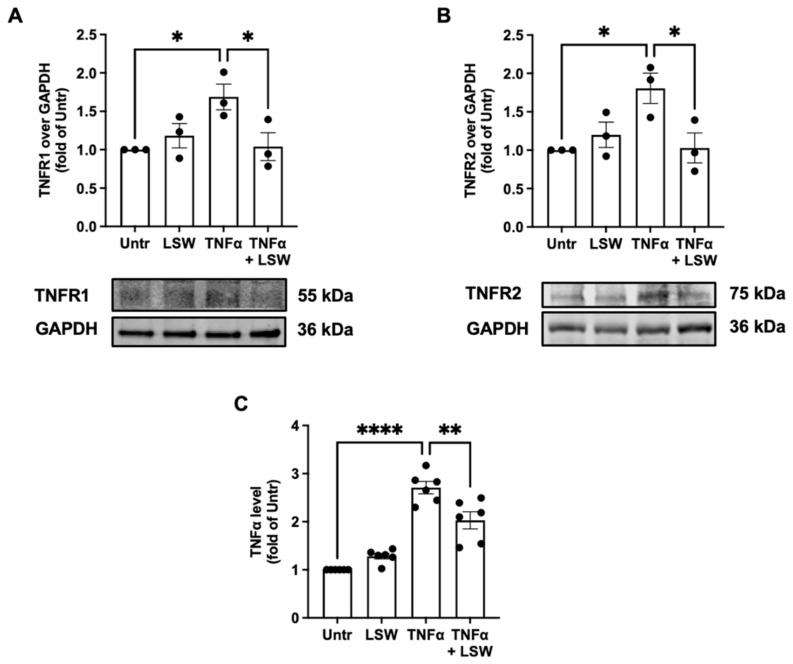
Effect of LSW treatment on TNFR1, TNFR2, and endogenous TNFα level in EA.hy926 cells. The cells were treated with LSW (50 μM) for 18 h before TNFα (10 ng/mL) stimulation for 6 h. Protein bands of TNFα receptor 1 (TNFR1, (**A**)) or 2 (TNFR2, (**B**)) were normalized to GAPDH. (**C**) TNFα level (cell lysate) was measured using ELISA kit. Data were normalized to the untreated group (Untr). *, *p* < 0.05; **, *p* < 0.01; ****, *p* < 0.0001.

TNFα stimulates cellular responses via its two receptors, TNFR1 and TNFR2; therefore, effect of LSW treatment on the levels of TNFR1 and TNFR2 was assessed. TNFα stimulation increased the levels of TNFR1 and TNFR2 (*p* < 0.05), while LSW treatment significantly attenuated both levels (Figure 5A,B). Additionally, LSW treatment inhibited the endogenous formation of TNFα in EA.hy926 cells (*p* < 0.01) (Figure 5C).

### 3.6. LSW Attenuates Inflammation Partially Dependent on SIRT1 in TNFα-Stimulated EA.hy926 Cells

**Figure 6:**
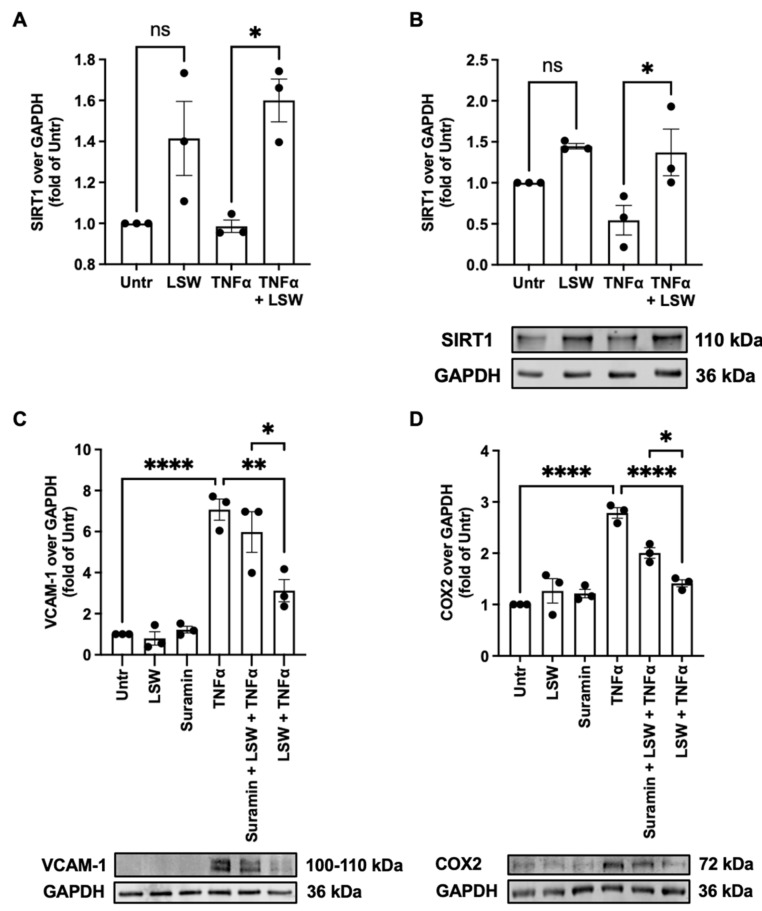
LSW attenuates TNFα-induced inflammation in EA.hy926 cells dependent on SIRT1. Panels (**A**,**B**) Effect of LSW treatment on SIRT1 expression. The cells were treated with LSW (50 μM) for 18 h before TNFα (10 ng/mL) stimulation for 6 h, followed by detecting SIRT1 mRNA (**A**) or protein (**B**) expression. Panels (**C**,**D**) Involvement of SIRT1 in LSW’s anti-inflammatory effect. The cells were treated with SIRT1 antagonist (suramin) for 30 min and co-treated with LSW (50 μM) for 18 h, before TNFα (10 ng/mL) stimulation for 6 h, followed by detecting the protein expression of VCAM-1 (**C**) and COX2 (**D**). Both mRNA and protein expression were normalized to GAPDH. Data were normalized to the untreated group (Untr). *, *p* < 0.05; **, *p* < 0.01; ****, *p* < 0.0001; ns, not significant.

SIRT1 is implicated in the regulation of endothelial dysfunction and cardiovascular diseases [20]. LSW treatment could enhance both mRNA and protein expressions of SIRT1 in EA.hy926 cells upon TNFα stimulation (*p* < 0.05); it also increased SIRT1 expression at the basal level despite not being significantly (Figure 6A,B). Since LSW treatment upregulated SIRT1 expression, whether LSW exerted its anti-inflammatory activity dependent on SIRT1 upregulation was studied. Indeed, by using a SIRT1 antagonist, suramin, the reduced VCAM-1 and COX2 expressions by LSW were partially restored (*p* < 0.05) (Figure 6C,D).

## 4. Discussion

Sustained oxidative stress and inflammatory responses in the vasculature proceed endothelial dysfunction, thus contributing to the incidence of type 2 diabetes, vascular remodeling, hypertension, atherosclerosis, and other cardiovascular diseases [21,22]. Therefore, the amelioration of oxidative and inflammatory statuses has been considered a key preventive strategy for combating various chronic diseases [23,24,25,26]. EA.hy926 is a somatic hybrid human umbilical vein cell line and has widely been used to evaluate the cytoprotective effect of various bioactive compounds on vascular oxidative stress and inflammatory responses [27,28,29].

Excessive production of reactive oxygen species (ROS), e.g., superoxide and hydroxyl ion, is the leading cause of oxidative stress, which then induces cellular damage and interrupts normal cellular functions [30,31,32]. Our findings show that LSW treatment reduced TNFα-stimulated oxidative stress, endorsed by the significantly lowered cellular superoxide and MDA levels (*p* < 0.0001) (Figure 2). Furthermore, the antioxidant effect of LSW was possibly independent of Nrf2 signaling, the major cellular antioxidant defense mechanism [33]; this was also confirmed by the unchanged cellular levels of superoxide dismutase 2 and glutathione peroxidase 4 after LSW treatment. These results suggest that LSW exerted antioxidant effect likely as a direct ROS scavenger. Indeed, among the three constituting amino acids of LSW, tryptophan is a strong antioxidant amino acid [34]. Additionally, the simultaneous presence of N-terminal hydrophobic amino acids, e.g., leucine, could further enhance a peptide’s antioxidant activity, which has previously been reported for many other peptides, such as LY (derived from rapeseed), LRW (pea), VGINYW (bovine α-lactalbumin), VRY, LKY, and VVHPKESF (chicken muscle) [17,35,36,37]. Serine is an amino acid with low antioxidant activity [34]. Despite being reported to reduce oxidative stress in human endothelial cells, its activity can only be exerted when its concentration reaches above 100 µM. Hence, serine was not considered as a major contributor to LSW’s antioxidant activity in the present study (50 µM of LSW used) [38]. Taken together, these findings demonstrated that LSW exerted its antioxidant effect in EA.hy926 cells possibly owing to the two terminal amino acid residues.

VCAM-1 and COX2 are two inflammatory mediators in endothelial cells [19,39,40]. LSW treatment significantly reduced TNFα-stimulated expression of VCAM-1 (*p* < 0.001) and COX2 (*p* < 0.01) in EA.hy926 cells (Figure 3). TNFα activates inflammatory responses via NF-κB and p38/JNK MAPK signaling, which differentially regulates the release of various inflammatory mediators/cytokines [19,41]. For example, VCAM-1 expression is mainly mediated by NF-κB signaling, while COX2 expression is dependent on both p38 and JNK signaling in endothelial cells [42]. Upon this, the present results suggest that LSW treatment inhibited VCAM-1 expression via NF-κB signaling and COX2 expression via both p38 and JNK signaling in EA.hy926 cells (Figure 4). Previously, a chicken-derived peptide, VVHPKESF, also inhibited TNFα-induced COX2 expression in EA.hy926 cells but only via suppressing p38 signaling [19]. Peptides from plant sources including soybean have been reported to regulate inflammatory responses via modulating intracellular signaling including NF-κB, MAPK, or adenosine monophosphate-activated protein kinase, both in vitro and in vivo [43,44,45,46]. To understand the mechanisms underlying the anti-inflammatory activity of LSW, the level of two TNFα receptors, TNFR1 and TNFR2, was also determined, since TNFα induces inflammation via these two receptors [47]. To date, bioactive peptides have rarely been reported to exert protective effects on cells against TNFα stress at the receptor level, except VVHPKESF, which has recently been reported to mitigate TNFα-induced inflammation and oxidative stress via suppressing TNFR1 signaling [19]. The exogenous addition of TNFα induced the expression TNFR1 and TNFR2 (*p* < 0.05) as well as the release of endogenous TNFα (*p* < 0.0001), which, however, were significantly inhibited by LSW treatment (*p* < 0.05) (Figure 5). These results demonstrate that LSW treatment attenuated TNFα-stimulated inflammatory responses via downregulating the loop of TNFα/TNFR1/TNFR2, indicating that LSW possibly inhibited TNFα signaling possibly also at the receptor level, in addition to the modulation of intracellular signaling.

SIRT1 belongs to the family of sirtuins, a family of nicotinamide adenine dinucleotide-dependent histone deacetylases [48]. SIRT1 activation protects various types of cardiovascular diseases. For example, SIRT1 stabilizes atherosclerotic plague and prevents cardiac ischemia and hypertrophy during aging, protecting cardiomyocytes [49]. SIRT1 protects endothelial dysfunction triggered by oxidative stress and inflammation [20]. For example, SIRT1 activation antagonizes inflammatory responses in response to various stimuli including lipopolysaccharides, interleukin-1 beta, transforming growth factor β, and others [50,51,52,53]. Under TNFα stress, LSW treatment upregulated both SIRT1 gene and protein expression in EA.hy926 cells (*p* < 0.05); protein expression of SIRT1 in the resting state was also enhanced but was not significant (*p* > 0.05) (Figure 6). Hence, whether or not LSW mitigated TNFα-induced inflammation via SIRT1 activation was further studied by using suramin, a SIRT1 antagonist; the contribution of SIRT1 to the antioxidant activity of LSW was not assessed because LSW reduced oxidative stress as a direct ROS scavenger as discussed previously. It was observed that antagonizing SIRT1 significantly attenuated the anti-inflammatory activity of LSW (*p* < 0.05), demonstrating that LSW-induced SIRT1 activation was partially involved in its anti-inflammatory action in TNFα-stimulated EA.hy926 cells (Figure 6). To date, very few peptides have been reported to activate SIRT1, via which their bioactivities were exerted. Only a soybean-derived tetrapeptide, Val–His–Val–Val (VHVV), and another dipeptide derived from potato, Ile–Phe (IF), have recently been reported to mitigate inflammation via SIRT1 activation in spontaneously hypertensive rats [54,55]. Others, including three egg-derived peptides, IRW, GWN, and GW, and a potato-derived peptide, DIKTNKPVIF, could enhance mitochondrial health both in vitro and in vivo dependent on SIRT1 activation [56,57,58]. Our results demonstrate the involvement of SIRT1 in the anti-inflammatory effect of LSW, further suggesting that SIRT1 might be a potential target of bioactive peptides underlying their biological effects. The mechanisms underlying SIRT1 upregulation by LSW treatment require further investigation in the near future. The bioactive impact of LSW might involve activation of AMP-activated protein kinase and more upstream signaling, as well as the connection between upregulation of SIRT1 and downregulation of NF-κB and MAPK signaling [59].

## 5. Conclusions

**Figure 7:**
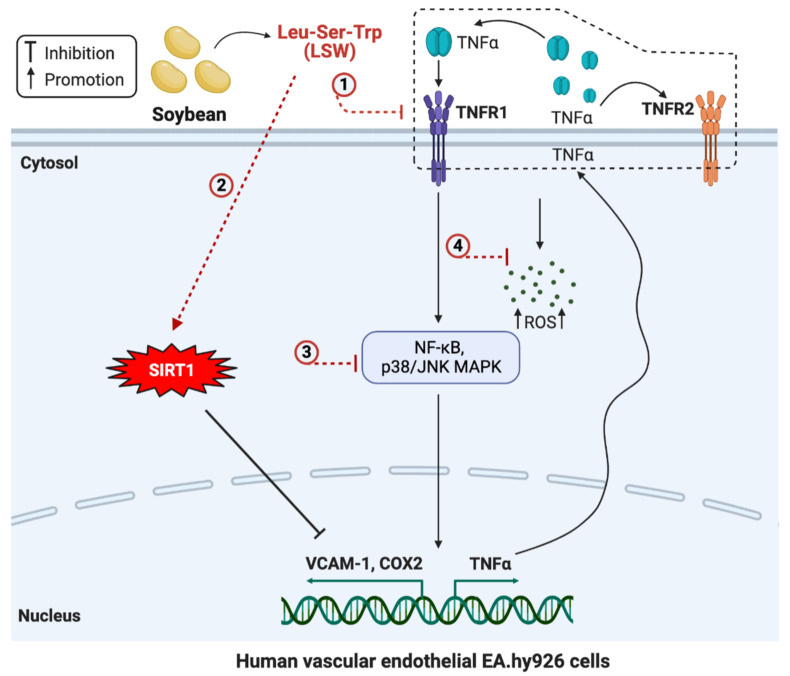
Mechanisms of action of LSW in mitigating TNFα-induced inflammation and oxidative stress in EA.hy926 cells. LSW might promote or inhibit cellular biological effects directly or indirectly (dash line, not necessarily needed to enter the cells).

In this study, the antioxidant and anti-inflammatory effects of a soybean-derived tripeptide, LSW, were evaluated in TNFα-stimulated EA.hy926 cells. LSW inhibited the TNFα-induced formation of superoxide and MDA, which was due to its free radical-scavenging ability. Additionally, LSW mitigated inflammatory responses (lowered VCAM-1 and COX2 expression) partially via SIRT1 and also via downregulating the TNFα/TNFR1/TNFR2 signaling system. Figure 7 summarizes the mechanisms of LSW underlying its antioxidant and anti-inflammatory activities. The present findings indicated that LSW can be a promising peptide-based functional food ingredient or nutraceutical for ameliorating endothelial inflammation and oxidative stress, which potentially contributes to the development of vascular dysfunction, hypertension, and cardiovascular diseases.

## Data Availability

Data available upon reasonable request from the authors.
